# The Role of Deep Learning and Gait Analysis in Parkinson’s Disease: A Systematic Review

**DOI:** 10.3390/s24185957

**Published:** 2024-09-13

**Authors:** Alessandra Franco, Michela Russo, Marianna Amboni, Alfonso Maria Ponsiglione, Federico Di Filippo, Maria Romano, Francesco Amato, Carlo Ricciardi

**Affiliations:** 1 Department of Electrical Engineering and Information Technology, University of Naples Federico II, 80125 Naples, Italy; alessandra.franco@unina.it (A.F.); michela.russo2@unina.it (M.R.); alfonsomaria.ponsiglione@unina.it (A.M.P.); mariarom@unina.it (M.R.); framato@unina.it (F.A.); 2 Department of Medicine, Surgery and Dentistry, Scuola Medica Salernitana, University of Salerno, 84081 Baronissi, Italy; mamboni@unisa.it (M.A.); federicodifilippo@hotmail.it (F.D.F.)

**Keywords:** Parkinson’s disease, deep learning, gait analysis, wearable sensors, video motion capture, human pose estimation, convolutional neural network

## Abstract

Parkinson’s disease (PD) is the second most common movement disorder in the world. It is characterized by motor and non-motor symptoms that have a profound impact on the independence and quality of life of people affected by the disease, which increases caregivers’ burdens. The use of the quantitative gait data of people with PD and deep learning (DL) approaches based on gait are emerging as increasingly promising methods to support and aid clinical decision making, with the aim of providing a quantitative and objective diagnosis, as well as an additional tool for disease monitoring. This will allow for the early detection of the disease, assessment of progression, and implementation of therapeutic interventions. In this paper, the authors provide a systematic review of emerging DL techniques recently proposed for the analysis of PD by using the Preferred Reporting Items for Systematic Reviews and Meta-Analyses (PRISMA) guidelines. The Scopus, PubMed, and Web of Science databases were searched across an interval of six years (between 2018, when the first article was published, and 2023). A total of 25 articles were included in this review, which reports studies on the movement analysis of PD patients using both wearable and non-wearable sensors. Additionally, these studies employed DL networks for classification, diagnosis, and monitoring purposes. The authors demonstrate that there is a wide employment in the field of PD of convolutional neural networks for analyzing signals from wearable sensors and pose estimation networks for motion analysis from videos. In addition, the authors discuss current difficulties and highlight future solutions for PD monitoring and disease progression.

## 1. Introduction

Parkinson’s disease (PD) is the second most common neurodegenerative disease (NDD) after Alzheimer’s disease [1]. The incidence of PD varies worldwide from 5 to over 35 new cases per 100,000 people per year, and the prevalence of the disease in industrialized countries is approximately 0.3% [2]. PD is characterized by a reduction in the number of dopaminergic neurons, which are those that produce dopamine [3], and the loss of this neurotransmitter is the main cause of movement disorders that make patients manifest symptoms such as bradykinesia, tremor, rigidity, and balance problems. Moreover, PD patients show gait dysfunctions, including a shorter stride length, shuffling steps, and difficulty initiating walking or turning [4,5]. In addition, most PD patients also show non-motor symptoms, such as depression, anxiety, sleep disorders, and cognitive difficulties, which tend to increasingly reduce their autonomy as the disease progresses [6,7]. Clinical scales are commonly used by neurologists to assess and quantify the disease state and progression, with the Movement Disorder Society-Unified Parkinson’s Disease Rating Scale (MDS-UPDRS) being the most widely employed [4,8]. Specifically, the MDS-UPDRS Part III assesses motor signs using 33 items and provides a comprehensive score that ranges from 0 to 132 [4,9]. However, these scores, although based on standardized procedures, may be biased by a subjective assessment of the raters. Several studies suggest that gait quantitative assessment can be conducted for both clinical and research purposes [10,11]. In many healthcare and scientific research institutions, gait analysis is used for diagnosis, evaluation, or monitoring the outcomes of a treatment [10,12,13]. In the clinical context of parkinsonism, gait analysis has been extensively employed to provide quantitative parameters that characterize the movement of patients with PD [14,15,16,17,18]. Indeed, some parameters in PD, like the cadence, velocity, and stride length, are specific and become worse during the disease course [19,20,21,22]. The human gait is the result of the correct and dynamic integration of voluntary and automatic movements. Various components are utilized for the objective measurement and analysis of the gait cycle: spatio-temporal features are the most commonly studied and refer to the global gait cycle or to the stride cycle; kinematic variables describe the joint angles and body segment orientation during walking in the sagittal, horizontal, or frontal plane; and kinetic features describe the forces and their effects on motion [23,24].

Traditionally, common methods for tracking human movement required a laboratory environment and the attachment of markers or sensors to segments of the body. Currently, the technologies fall into two main categories: wearable and non-wearable systems. Wearable systems utilized for gait analysis include inertial, pressure, and force sensors [25,26,27,28], while some examples of non-wearables are ground sensors and vision-based technologies (such as single or multiple cameras and stereoscopic vision) [17,29].

**Figure 1:**
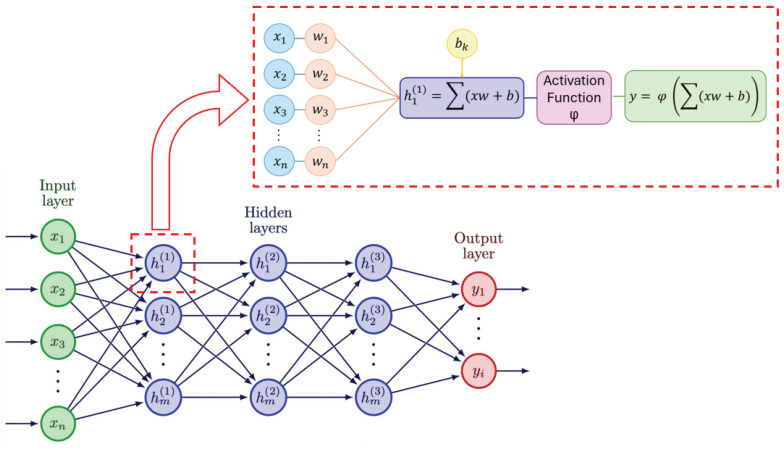
Composition and working mechanism of DNN.

In the study of PD, deep learning (DL) techniques have been increasingly employed to analyze different aspects of the disease [30,31,32]. In particular, the detection of characteristic movement patterns typical of PD include the monitoring of symptoms using data from wearables or cameras, such as tremor, rigidity, and freezing of gait (FoG) [33,34]. In Figure 1, we can observe the composition of a typical deep neural network (DNN) with all its layers: input, hidden, and output. When we examine a single neuron (as shown in the zoomed-in section), we see that it receives inputs from the previous layer, multiplies them by their respective weights, adds the biases, and sums everything. The result is then passed through an activation function, which produces the output that is sent to the next layer. This process is repeated for all the neurons in all the layers of the network.

In addition, by analyzing longitudinal data collected over time, deep learning (DL) has been employed to predict symptoms deterioration and disease progression, thus enabling timely and personalized intervention [35]. A common approach is to implement DL models, such as a convolutional neural network (CNN) to analyze the gait data obtained from wearable and non-wearable technologies [33,36,37]. These sensors can capture small changes in movement dynamics associated with PD symptoms. By feeding this data into a CNN, the model can learn to automatically extract relevant features from the gait signals, such as the stride length, walking speed, and variability in movement patterns [38].

Moreover, the literature has shown a growing interest in computer-vision-based motion analysis, where it has mainly focused on the automatic diagnosis of PD [39,40,41,42]. Video-based motion analysis deals with the development of algorithms and techniques for the automatic interpretation of images or videos to extract information regarding human movement. Human pose estimation (HPE) is an important sub-category of computer vision that focuses on identifying and tracking the positions of joints or parts of the human body in images or videos from data acquired by single or multiple cameras [22,42,43].

The present review aimed at investigating the role of DL in the motion analysis of PD. Specifically, it sought to present the main DL techniques currently implemented in the literature, with particular attention to the characteristics of the population studied; the devices used to acquire motor data; and the primary artificial intelligence techniques operated for disease prediction, monitoring, and diagnosis.

## 2. Materials and Methods

This systematic review adhered to the guidelines described in the PRISMA guidelines [44] (the checklist is included in the Supplementary Materials). This research was conducted using three databases: Scopus, PubMed, and Web of Science. The search query, which was formulated using the PICO strategy [45], incorporated “Deep learning” AND (“gait analysis” OR “motion analysis”) AND (“Parkinson’s disease” OR “parkinsonism”), which was applied to search within the titles and abstracts of selected databases. The outcome was intentionally omitted as a keyword to maintain a broad scope for the query. The range of publication time considered six years, from 2018 (which is the year of the first paper published on this topic) to 2023. The last group of scientific papers appeared in the year 2024. The articles were included and excluded in the analysis according to the following criteria of eligibility.

The selection criteria were as follows:

- Focus on PD with no known cause (idiopathic PD);
- Gait analysis with a clear description of the experimental protocol to study PD;
- Discussion on the details of the neural network used and the performance indices utilized.

The exclusion criteria were as follows:

- Non-English articles, book chapters, and reviews;
- Articles with unavailable full text;
- Articles related to a healthy control (HC);
- Articles unrelated to DL;
- Articles with an absence of focus on PD and an absence of gait analysis.

The researchers created a summary table that aggregates the data of the selected articles. The information collected in the final extraction included the title, journal, year of publication, sample characteristics (size and type of population studied), type of dataset, study objective, instrumentation, methodology, tasks, gait variables, and study results. The summary of included articles were collated and synthesized to identify common themes, new technologies, limitations, and future research directions.

**Figure 2:**
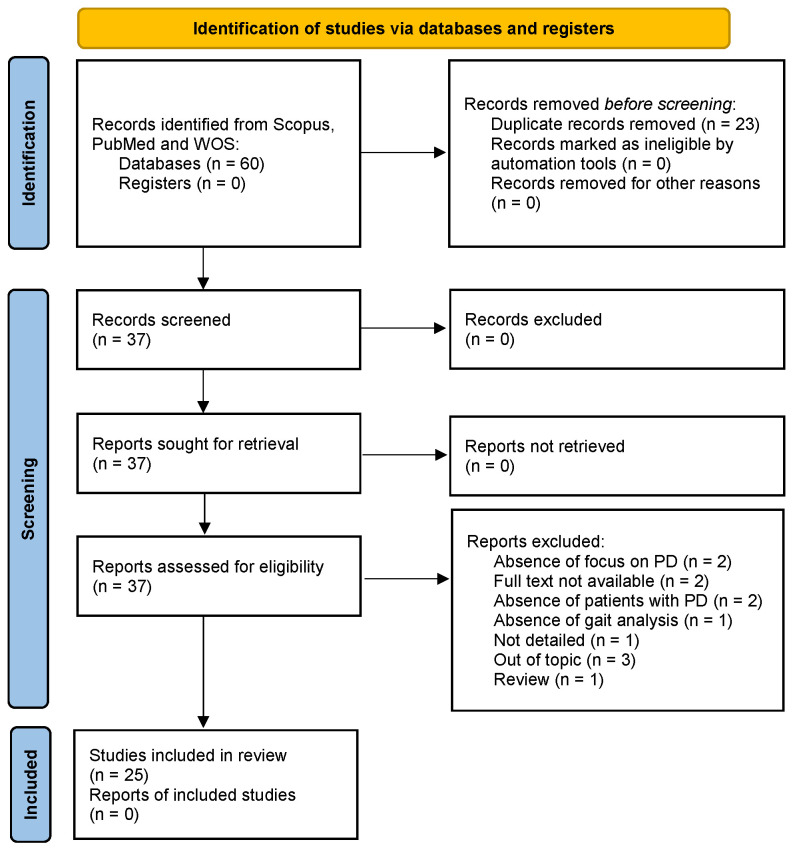
Workflow for the inclusion of the articles.

The study selection process followed the PRISMA flowchart. In Figure 2, we illustrate this review’s workflow.

A total of 31 potential articles were analyzed from the research on Scopus, 9 on PubMed, and 20 on Web of Science. After removing 23 duplicates, we excluded 12 articles after applying the eligibility criteria and conducting a thorough analysis of the full text. Overall, 25 articles were included in our study. Each identified record was independently reviewed by two reviewers to determine its eligibility based on the inclusion and exclusion criteria. The reviewers worked independently without consulting each other during this initial phase to reduce bias and ensure the objectivity of the screening process. In the case of disagreement between the two reviewers regarding the eligibility of a record, a third reviewer was consulted, who examined the contested records and provided the final decision on which studies to include in the review.

## 3. Results and Discussions

### 3.1. Characteristics of the Included Studies

**Figure 3:**
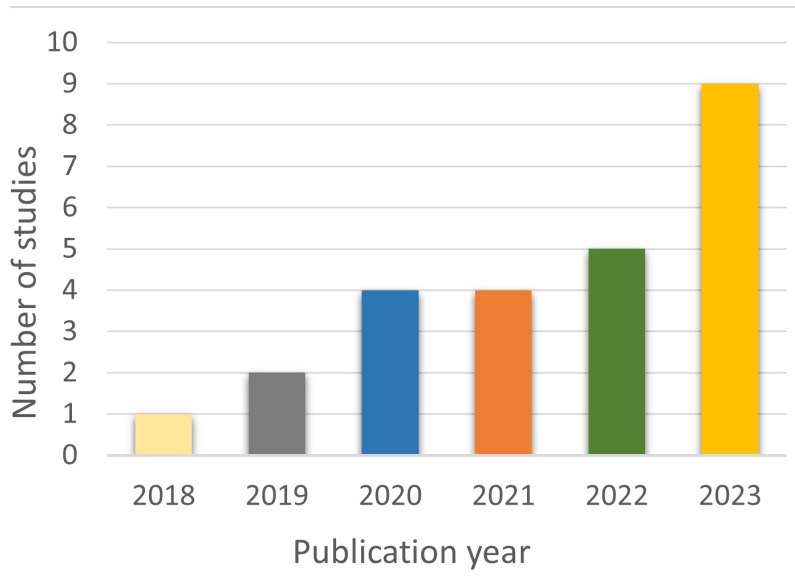
Temporal trend of publications on PD and DL with gait analysis.

As shown in Figure 3, there was an increase in the publication of studies on the topic from 2018 to 2023, and the majority of them were published in 2023.

**Figure 4:**
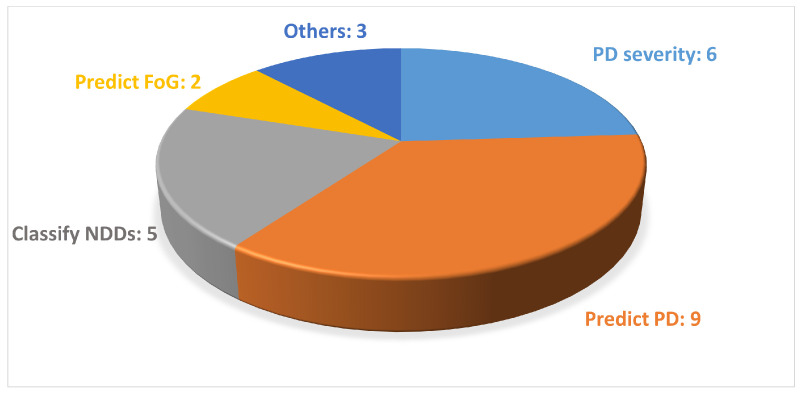
Distribution of the studies found according to the objective of this study. In this figure, NDDs stands for neurodegenerative diseases.

**Figure 5:**
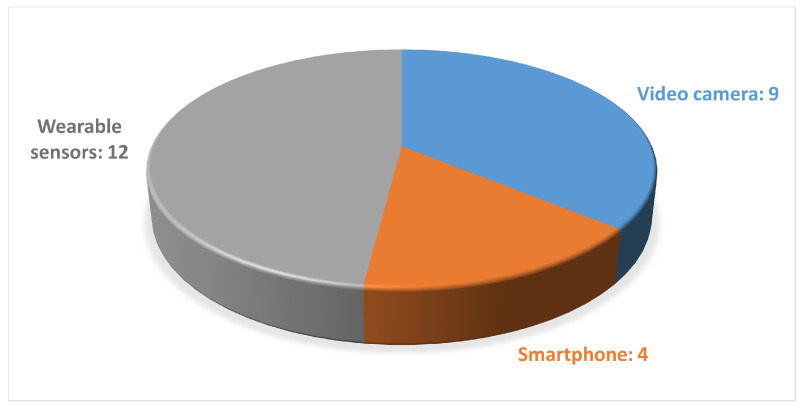
Distribution of the studies found regarding gait-acquisition devices.

The analyzed studies mainly focused on predicting PD, while various data acquisition devices, including wearable sensors and markerless motion capture systems based on both a single camera or a smartphone, were used to collect gait data, as shown in Figure 4 and Figure 5.

Among the studies that investigated the use of smartphones as an acquisition device, only Abujrida et al. utilized the accelerometer and gyroscope sensors by treating the device as a wearable sensor, while others employed the smartphone’s camera to capture videos [46].

### 3.2. Characteristics of the Datasets

**Figure 6:**
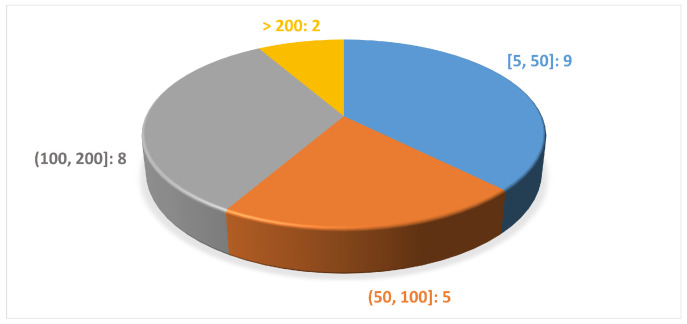
Distribution of the studies according to the number of subjects per study.

The number of subjects involved in the analyzed studies ranged from a minimum of 9 to a maximum of 456 subjects, as shown in Figure 6. Moreover the subjects were aged between 40 and 75 years and there was a greater number of men compared with women, as we expected from previous studies on the disease [47,48].

### 3.3. Experimental Approaches Adopted

In the context of this review, two different methods for gait data acquisition were found, based on which the authors made different choices about the typology of a DNN to adopt in their experiments. Indeed, in some cases, wearables, such as accelerometers or gyroscopes, were exploited, and the resulting dataset consisted of signals acquired from the sensors; in other cases, the authors used cameras to capture walking videos, which resulted in datasets composed of images extracted from the videos. In a few cases, smartphones were considered as a wearable by exploiting the built-in accelerometer and gyroscope, and in other cases for video acquisition. This motivated us to compare the articles based on the data acquisition method analyzed. Therefore, we analyzed the articles in which wearable sensors were used and those that explored a markerless motion capture system based on video.

#### 3.3.1. Wearable Sensors

**Figure 7:**
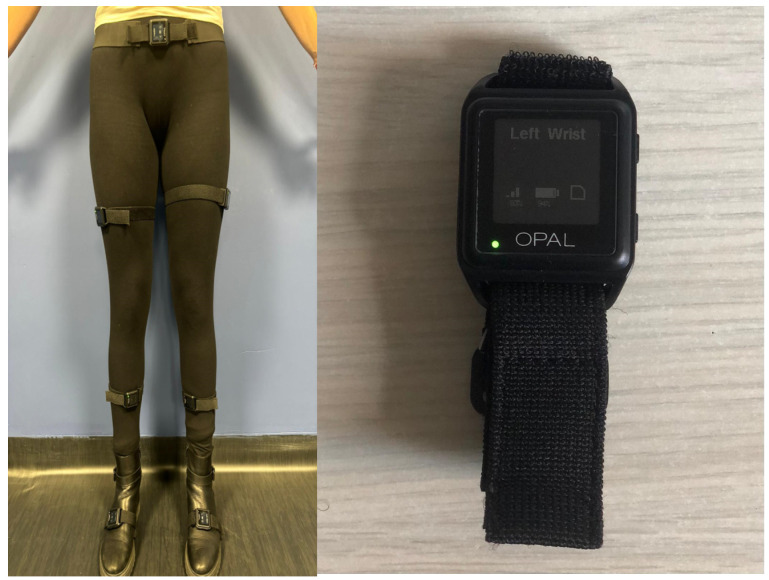
On the left, classic positioning of wearable sensors on the lower limbs to calculate joint kinematics is shown; on the right, an example of the wearable sensor APDM Opal™ (https://share.apdm.com/documentation/MobilityLabv1UserGuide.pdf (accessed on 4 April 2024), APDM, Portland, OR, USA), which is composed of two accelerometers, one gyroscope, and one magnetometer that records in three axes (vertical, mediolateral, anteroposterior), is shown.

Table 1 shows the articles that employed wearable sensors to investigate gait analysis in PD. Figure 7 shows an example of wearable sensors.

**Figure 8:**
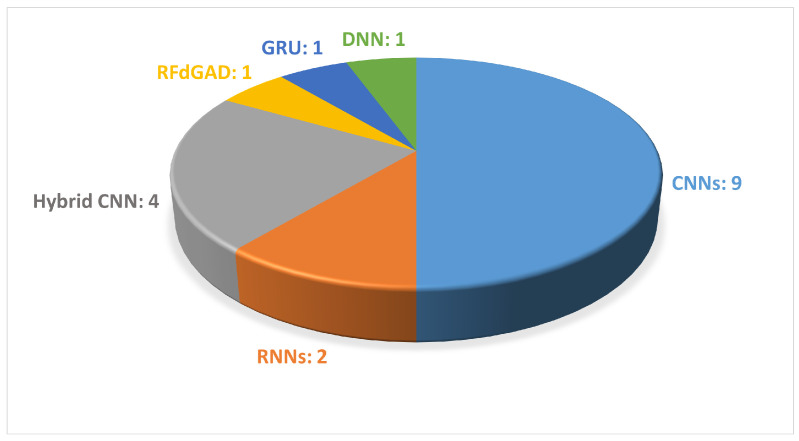
The most common DL-based approaches used in wearable-sensor-based gait analysis are presented. The figure shows the names of the DL methods and the number of articles that used each method.

It can be observed that 13 out of 24 studies extracted the information to describe gait from signals obtained from wearable sensors. The employed features included the magnitude vectors of the acceleration and rotation, force domain, peak domain (mean, standard deviation, max, and min values of peak data) and abnormality domain, lengths of the stance and swing phases (for left and right), and time–frequency spectrograms. Figure 8 illustrates the DL-based approaches employed in the reviewed papers.

In 2023, Abujrida et al. used smartphone accelerometers and gyroscopes to collect data from 456 subjects (152 PD and 304 HC subjects). The objective was to determine whether PD patients took their medication by utilizing the DeepaMed CNN model [46]. The study findings suggest that medication non-adherence among PD patients can be reliably predicted through the smartphone-based monitoring of motor symptoms. In 2021, Peraza et al. adopted samples for steps and samples for strides recorded with four sensors: an AX3 (Comapy name: Axivity, address: Newcastle upon Tyne, United Kingdom (UK)) on a wrist, a GENEActiv (Company name: Activinsights Ltd, address: Kimbolton, Cambridgeshire, United Kingdom (UK)) on the other wrist, a AX3 attached with hypoallergenic tape to the lower back, and a GENEActiv sensor strapped around the shin. For the experiments, 12 subjects (6 PD and 6 HC subjects) performed slow gait, normal gait, fast gait, and timed up-and-go (TUG) tests [49]. The objective of this study was to extract temporal and spatial gait parameters from a single wearable triaxial accelerometer using two DNN models: a CNN and a two-layer DNN. They opted for a CNN with a U-Net architecture, where for the training datasets, the time series were divided into 512-sample segments with 128-sample hops. In both of the previous papers, the authors trained CNNs on private datasets. As shown in the table, Abujrida et al. achieved an accuracy of 98.2% and a precision of 97.7%, while Peraza et al. reached their goal of finding that the stance time and stride time were significantly different for the normal gait task and the Pearson correlation coefficients were greater than 0.75 for the spatial parameters.

Three articles among those that used wearables aimed at distinguishing between various NDDs using gait data. Specifically, the diseases to be identified included PD, Huntington’s Disease (HD), and amyotrophic lateral sclerosis (ALS) [50,51,52]. The authors of all three articles used gait data from 16 HC subjects, 13 patients with ALS, 20 patients with HD, and 15 patients with PD obtained from the PhysioNet Gait Dynamics in NDDs to validate their algorithms. This database contains two types of data, that is, raw force series data and a derived time series from the raw data. In 2023, Erdaş et al. divided the problem into subsets that comprised four classes: PD, HD, ALS, and a control group [50]. Furthermore, subgroups were created by comparing each disease individually against the control group, along with a disease versus control subgroup, where all diseases were grouped under a single label. To assess the disease severity, each condition was further subdivided into categories, and distinct machine learning (ML) and DL techniques were employed to address the prediction task separately for each subgroup. In this paper, multi-layer perceptron, random forest, extra trees, and k-nearest neighbor methods were employed as classification methods, while voting, stacking, and one-dimensional CNN methods were used as regression methods, in order to predict different neurodegenerative diseases.

In 2020, Lin et al. proposed a DL approach to classify different NDDs based on the recurrence plot of vertical ground reaction force (vGRF) data [51]. In particular, a pre-trained AlexNet CNN was utilized from MATLAB R2018a DL ToolboxTM in this system. Also, Setiawan et al. used a pre-trained AlexNet CNN; however, the time-domain vGRF signal was modified into a time–frequency spectrogram by means of a continuous wavelet transform [52]. Then, feature enhancement with principal component analysis was considered; among these, the best performance in terms of accuracy, sensitivity, specificity, and area under the curve (AUC) was achieved by Lin et al. Specifically, they obtained an accuracy greater than 95%, sensitivity and specificity greater than 90%, and an AUC greater than 90% [51]. Indeed, the two articles published in the following years were unable to overcome the performance obtained with the adopted neural network employed by Lin et al. with the same dataset and for the same purpose.

All the other reviewed studies that employed wearables aimed at differentiating between individuals affected by PD and the HCs. Vásquez-Correa et al. and Carvajal et al. used time–frequency spectrograms and spatio-temporal features obtained with Embedded Gait analysis using Intelligent Technology (eGaIT system (Company name: Hasomed GmbH, address: Magdeburg, Saxony-Anhalt, Germany)), which consists of sensors attached to the patient’s shoes [53,54]. In 2019, Vásquez-Correa et al. proposed a methodology to model the difficulties to start or stop movements considering information from speech, handwriting, and gait [53]. The neurological conditions of patients were assessed across various stages of the disease: initial, intermediate, and advanced stages. Additionally, they examined the resilience of the proposed approach by incorporating speech signals from three distinct languages: Spanish, German, and Czech. The authors performed three main experiments: classification of PD patients and HC subjects, classification of PD patients in different stages of the disease according to the total MDS-UPDS-III score, and classification of PD patients in different stages of the disease according to specific impairments in the lower and upper limbs and in speech by considering sub-scores of the MDS-UPDRS Part III scale. Individual CNNs were trained for each modality on a cohort of 84 subjects (44 PD patients); afterward, multimodal assessment was performed by combining the three bio-signals.

In 2022, Carvajal et al. acquired gait data from 134 subjects (68 PD and 66 HC subjects) and considered two subgroups of HCs, namely, elderly and young [54]. This was the first study in gait analysis where temporal and spectral information was combined in an architecture of DL. They employed segments of 3 s of raw time series data to train three different DL architectures: CNN, gated recurrent unit (GRU), and CNN + GRU. The authors validated that the combination of CNN and GRU methods yielded results comparable with those obtained with GRUs alone. However, due to the small dataset employed in this study, it was not possible to demonstrate the authors’ starting assumption that the combination of CNN and GRU would lead to greater accuracy. The authors of the previous articles thus trained their neural networks on the same cohort of subjects with the shared goal of predicting PD. However, Vásquez-Correa et al. achieved superior results, where they reached an accuracy of 97.6% and an AUC of 98.8% by merging the three analyzed signals [53].

Ma et al., Zhong et al., Aşuroğlu et al., Setiawan et al., Xia et al., and El Maachi et al. used the public dataset Physionet Gait in PD (93 PD patients and 73 HC subjects) in their studies, which contains gait signals collected via sixteen pressure sensors placed on the left and right soles of the subjects’ shoes, with eight on each foot [55,56,57,58,59,60]. In 2023, Ma et al. proposed an explainable learning architecture to address the challenge of early PD diagnosis by analyzing differences between PD patients and healthy individuals. The study incorporated three domains (force, peak, and abnormality) to extract twenty-two features for analysis [55]. Subsequently, the ANOVA with recursive reduction (ARR) was first used to determine which features should be removed; then two neural networks, XGBoost and CNN, were trained. They found that the learning model increased the accuracy of the performance analysis. In the same year, Zhong et al. proposed a robust frequency-domain-based graph adaptive network (RFdGAD) for PD detection from gait information. Previously existing DL methods for PD detection did not take into account automatic feature extraction in the frequency domain [56]. The RFdGAD utilized initially learns frequency-domain features of signals from individual foot sensors using a frequency representation learning (FRL) block. Subsequently, it employs a graph adaptive network block (GAD) that takes frequency-domain features as the input to dynamically learn and leverage the connections between various sensor signals, and thus, enhance the PD detection accuracy.

Aşuroğlu et al., Setiawan et al., Xia et al., and El Maachi et al., in addition to the prediction of PD, also considered the severity of symptoms as an objective [57,58,59,60]. In 2022, Aşuroğlu et al. subjected the signal data from the sensors to feature extraction. These features were then input into a hybrid DL architecture that combined CNNs with locally weighted random forests [57]. The study of Setiawan et al. in 2021 aimed at extracting pattern features and visualizations from vGRF signals in PD patients across different severity stages (0, 2, 2.5, and 3 on the HY rating scale) [58]. The authors achieved their objectives by transforming one-dimensional time-domain signals into two-dimensional patterns (images) using a feature transformation method based on a continuous wavelet transform. Subsequently, they constructed and trained a CNN classifier and evaluated the performance of the classification algorithm through cross-validation. Previously, Xia et al. (2020) separately modeled the left and right gait parameters using a CNN, followed by an attention-enhanced long short-term memory (LSTM) network [59]. However, the first algorithm to perform a severity prediction based on the UPDRS, in addition to classifying each subject’s walk either as Parkinson’s disease or a control, was the one proposed by El Maachi et al. in 2020 [60]. Each subject’s walk was divided into smaller segments of 100 time steps with 50% overlap, and these segments were then used to train a 1D CNN (1D-Convnet) so that it was able to classify them. Among the studies that primarily aimed to predict PD, Xia et al. achieved the best performance in 2020, where they reached an accuracy of 99.31%, a sensitivity of 99.35% and a specificity of 99.23% with their CNN-LSTM [59]. These numbers were not outperformed by the neural networks trained by other authors. Regarding the goal of predicting the severity of PD, Aşuroğlu et al. had the highest results in 2022, with an accuracy of 99.5%, a sensitivity of 98.7%, and a specificity of 99.1% [57].

In general, the methods exploited for analyzing data from wearable sensors employ CNNs and DNNs, such as U-Net and AlexNet, and other ML techniques, like random forest and k-nearest neighbor. These techniques have enabled authors to extract and analyze temporal and spatial features from accelerometer and gyroscope data, and thus, demonstrate high accuracy in diagnosing neurodegenerative diseases, such as PD, HD, and ALS, despite the limitations of small datasets. Additionally, the use of signal transformations, like a continuous wavelet transform, has improved the performance of predictive models.

#### 3.3.2. Vision-Based Motion Capture System

Table 2 shows the studies that employed video capture to study gait analysis in PD.

**Figure 9:**
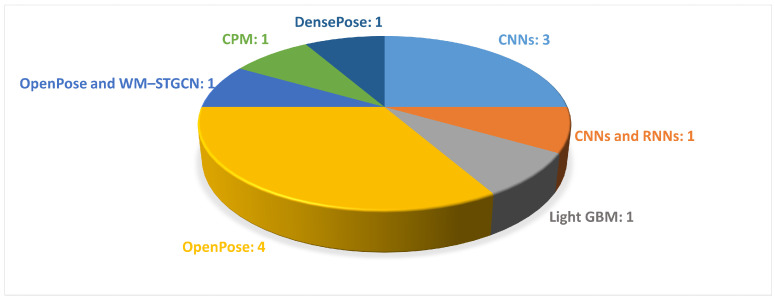
The most common DL-based approaches used in video-based gait analysis are presented. The figure shows the names of the DL methods and the number of papers that employed each method.

As shown in Table 2, vision-based motion analysis were employed in 12 studies of this systematic review to extract information from sequential images in order to describe movement. The variables of gait considered in the following studies mainly revolved around the gait spatial parameters; the oscillatory movements of the upper limbs; and the movements in the sagittal plane of the lower limbs, such as knee flexion and ankle dorsiflexion. In particular, the maximum and minimum flexion and extension for the respective angle joints were computed. Figure 9 illustrates the DL-based approaches employed in the reviewed studies.

In 2023, Eguchi et al. exploited video data recorded during gait assessment to predict the severity of motor symptoms, which involved 74 PD patients [61]. The videos of all participants were divided into three groups based on the UPDRS Part III scores assigned by neurologists: mild, moderate, and severe. Specifically, they employed the ECO-Lite CNN architecture to learn spatio-temporal features from the videos. Initially, sixteen frames from each video were processed using a 2D network to generate 96 feature maps. Subsequently, these maps were fed into a 3D network to analyze the interactions between frames. The authors calculated the coefficient of determination (R^2^) between those scores to evaluate the model’s goodness of fit. The R^2^ for the total UPDRS Part III score and the subscores of axial symptoms, i.e., bradykinesia, rigidity, and tremor, were 0.59, 0.77, 0.56, and 0.46, respectively.

**Figure 10:**
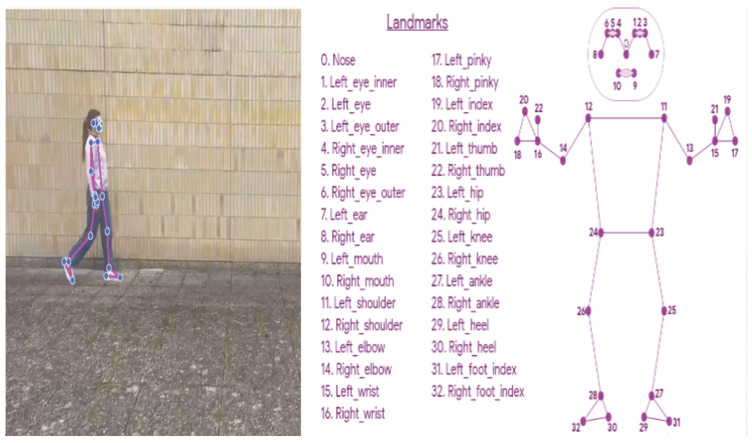
On the left, an example of the human pose estimation technique during video recording of an individual walking is shown; on the right, landmark positioning based on the skeleton approach is shown.

In the field of motion capture by videos, HPE has been widely used for the extraction of the body configuration in images or videos as a 3D markerless method against classical techniques and marker-based motion capture systems. Figure 10 shows an example of an HPE technique during a single walking test.

In 2021, Rupprechter et al. utilized videos captured with commercially available mobile phones as part of routine gait assessments to evaluate motor performance in PD patients [62]. They introduced a markerless pose estimation system designed to extract quantitative gait features relevant to PD. Key body points were extracted from each frame using the OpenPose library, from which six gait features were derived. These features encompassed critical aspects of movement, such as speed, arm swing, postural control, and movement smoothness. The study found a high correlation of 0.80 between the manually labeled and automatically estimated step frequencies.

Similarly, Zanela et al. introduced a smart system that utilized gait videos from 10 subjects, which consisted of 5 individuals with PD and 5 HCs. The system employed a stereoscopic device equipped with a pair of cameras mounted with co-planar optical planes and co-linear sensor bases, along with pose estimation techniques, to evaluate gait impairments and assess the disease burden [63]. In this preliminary study, the authors proposed a system that would be able to correctly recognize and qualitatively highlight the same alterations assessed by the MDS-UPDRS scores [63].

Additionally, Abe et al. compared peak-to-peak (P-P) data of left and right arm swings in PD patients using a gait analysis system based on OpenPose and video captured by a smartphone camera. They analyzed arm swing asymmetry (ASA) and P-P data from both arms and compared these metrics with the MDS-UPDRS scores. The system demonstrated an accuracy that ranged from 72.73% to 82.35% [64].

Similarly, Guayacan et al. proposed a markerless strategy for analyzing body segment kinematics to characterize PD during walking, which was captured in sagittal video sequences that used a single camera. They utilized DensePose to generate dynamic pose masks of specific body segments, such as the head, trunk, and limbs. Kinematic patterns were densely captured within each segmented region, and motion trajectories were computed to represent pixel-by-pixel displacement. The approach was validated using various pretrained classification models and evaluated on a dataset of 11 control subjects and 11 PD patients, where it achieved an average accuracy of 99.6% for the lower limbs and head regions [65].

Zhang et al. presented a novel method for recognizing the gait of PD patients by recording gait videos using the cameras of two smartphones [66]. Skeletal features were first extracted from the videos using OpenPose. They then applied a weighted adjacency matrix with virtual connection and multi-scale temporal convolution in a spatio-temporal graph convolution network graph convolutional neural network (WM-STGCN), which offers an efficient mechanism for directly learning joint trajectories. The experimental results demonstrated that the proposed method achieved a best accuracy of 87.1%.

Similarly, Li and co-workers [67] assessed the feasibility of extracting movement trajectories from videos of PD patients for detecting and estimating the severity of levodopa-induced dyskinesia by using the convolutional pose machine (CPM), which is a DL-based pose estimation algorithm. The CPM generated a 14-point skeleton, which included key joints, such as the head, neck, shoulders, elbows, wrists, hips, knees, and ankles, with joint trajectories extracted independently for each frame. They focused on 15 gait kinematic features to train an ML model, which achieved the best results, with an AUC of 0.930 for detection and a correlation coefficient of 0.661 for the severity estimation.

All these papers collectively explored the application of markerless pose estimation techniques for analyzing gait patterns in PD patients. They emphasized the use of video-based motion capture methods by employing markerless pose estimation systems, such as OpenPose, DensePose, and CPM to extract movement features. In the studies [62,63,64,65], videos served as the primary data source for assessing motor performance in PD patients. The authors demonstrated the effectiveness of computer vision techniques in capturing the nuanced movement patterns associated with PD achieving accuracy values of over 90% in identifying kinematic measurements of the lower limbs with DensePose and analyzing upper limb sway with OpenPose. In addition, several studies demonstrated that video-based gait analysis systems can effectively correlate with MDS-UPDRS scores, which provides valuable insight into motor symptom severity in PD patients, as shown by the results of [61,63,67].

Markerless motion capture systems were also employed for a common focus to analyze gait patterns, specifically in PD patients that experienced FoG symptoms. Sato et al. proposed a method for quantifying gait features and detecting FoG events by extracting the cadence from normal and parkinsonian gait movies recorded with a home video camera. Sequential gait features were obtained from the movies by extracting body joint coordinates using the OpenPose network [68]. In a similar way, Hu et al. proposed a promising novel graph convolutional neural network (GCNN) FoG detection method that involved collecting more than 100 videos from 45 PD patients in a frontal view with an accuracy over 89% [69].

Our systematic research revealed a common crucial point: understanding the distinctive characteristics of walking patterns associated with various neurodegenerative pathologies. Through the application of videos and DL algorithms, these studies delved into the analysis of walking videos to extract pertinent information for the quantification and automatic differentiation of these diseases.

In particular, Kaur et al. investigated the effectiveness of a vision-based model for predicting gait dysfunction in several neurodegenerative diseases, such as PD and multiple sclerosis, during single and dual trials. The study used two digital cameras, which were positioned to capture the subjects’ lower half and foot movements from the front and right side at a frame rate of 30 frames per second [70]. The OpenPose network was first used to locate 2D joint points for each subject, followed by a pose transformation into 3D space. By segmenting the gait steps and identifying heel strikes, several DL algorithms, such as a CNN and recurrent neural network (RNN), were trained and tested to classify the two neurodegenerative diseases. In the single task, the RNN produced the highest accuracy and AUC of 78.1% and 0.87, and the CNN had the highest accuracy of 75% in the dual trials.

Similarly, Gul et al. proposed a hybrid system based on a CNN and using videos of 28 patients taken from the front, back, and both sides during walking in order to distinguish PD from multiple sclerosis (MS) [71]. In the study, the data were analyzed using ML techniques and the best accuracy score was obtained as 87.5%.

In the same year, Iseki et al. studied the gaits of patients with PD and other neurological diseases while walking on a circular path approximately 1 m in diameter [72]. Participants were asked to walk clockwise for two laps and counterclockwise for two laps at their comfortable speed. The Three-Dimensional Pose Tracker for Gait Test (TDPT-GT) application, which is a markerless motion capture system based on ML, was used in combination with an iPhone camera to record and analyze each participant’s gait. Gait data were collected using a LightGBM model, which combines a gradient-boosting decision tree with gradient-based one-sided sampling and exclusive feature bundling.

## 4. Conclusions

PD has a significant impact on one’s quality of life by affecting various aspects of daily life, such as mobility, communication, autonomy, and mental health. Motor impairments that occur during the disease can be identified through an objective and quantitative gait analysis. This approach can improve clinical practice by aiding in diagnosis, symptom monitoring, rehabilitation, and fall prevention. Traditional methods of diagnosis and evaluation rely on clinical assessment, which can be subjective and imprecise, especially in the early stages of the disease. To overcome these limitations, numerous studies integrated gait analysis with ML and DL approaches to detect and classify PD and its severity [17,33,73,74,75,76,77]. Although various ML algorithms have been used to predict PD, a recent review by Landolfi et al. discussed and compared these approaches [33]. However, there remains a gap in the literature specifically addressing a comparison of DL algorithms used for similar purposes. For instance, Loh et al. conducted a comprehensive review on DL models for the automated identification of PD that covered a broad range of applications, including brain analysis and motor symptoms, such as handwriting and speech [78]. However, their review did not focus specifically on the integration of DL algorithms with tools for movement assessment.

Indeed, the aim of the present review was to present novel DL-based solutions for future research efforts focused on the study of gait patterns in patients with PD using different measurement tools. This includes applications for early disease detection, assessment of disease progression, and the implementation of therapeutic interventions. By using DL models, it was possible to thoroughly analyze motion data from wearable sensors or markerless techniques to identify characteristic patterns associated with PD. These models can process both temporal and spatial motion information, which allows for the detection of subtle changes in motor patterns over time. The topic on which this review focused is emerging, as there has been an increasing production of scientific publications on it from 2018 to 2023, and thus, this represents an initial phase of interest and development of the theme. Considering that 2018 was the first publication year, this confirmed the recent relevance and growing interest in the topic. The authors of the included papers frequently opted to utilize CNNs in their studies. Among the various available architectures, the use of AlexNet proved suitable when the features were time–frequency spectrograms. This choice was guided by AlexNet’s reputation for processing complex data through its deep structure and extracting relevant features from images. Although time–frequency spectrograms are not traditional images, they provide a visual representation of spatio-temporal data that can be effectively processed by CNN architectures, like AlexNet. Exploiting an AlexNet pre-trained on a large set of images, the authors achieved significant results by transferring knowledge from the network [51,52,58]. Moreover, ResNet has been particularly useful for the long-term analysis of gait signals. In this context, the presence of numerous residual structures has helped to capture complex relationships between motion signals and PD characteristics [58,70]. In the reviewed papers, we found a widespread utilization of wearables to acquire gait data. Among them, the most commonly utilized were ground force sensors, which are force-sensitive resistors mounted on an insole inside the shoe and powered by a battery. Accelerometers and gyroscopes mounted on shoes or even on the tibia, wrist, or lower back were also employed. In addition, we observed a greater utilization of private datasets in the context of video capture compared with wearables.

Overall, the reviewed papers extensively explored the use of video systems, such as a single or multiple cameras in combination with markerless pose estimation techniques for analyzing gait patterns in PD. In particular, markerless motion capture techniques were employed to compare the gait cycles of patients with PD with those of the HCs and to demonstrate that video-based gait analysis systems can effectively correlate with objective clinical scales. These techniques were also used to investigate specific motor symptoms, such as FoG, and to differentiate between various neurological disorders. It is clear from our systematic review that the markerless motion capture approach integrates smoothly into existing clinical practice, as video capture during the motion analysis of PD patients is already common practice [61,62,69,79]. However, it was not possible to define a unique positioning of the cameras. Indeed, different chamber arrangements were found in our research.

In some studies, a single camera was positioned either frontally or laterally to the walkway [61,64,65], whereas in studies that used multiple cameras, the cameras were positioned frontally, laterally, and even at 45° to the subject [63,65,66]. In the context of DL approaches for video analysis, CNNs are among the most frequently used methods, particularly when compared with RNNs; see Figure 9. In the field of HPE, OpenPose has been frequently used for the detection of key points of the human body within video footage, as shown in Figure 9. It facilitates the extraction of movements along the sagittal plane, including joint angle movements, such as knee, ankle, and hip flexion–extension, as well as upper limb balance. Additionally, RNNs have been employed to analyze temporal sequences of motion data, thus enabling the detection of subtle changes in gait patterns over time [70,80]. Furthermore, hybrid neural networks have been used, which combine multiple types of neural architectures to improve the performance of prediction models [54,59]. The analyzed articles applied various DL techniques to public datasets or their own datasets, which have highly variable dimensions. Moreover, it was found that some of the scientific studies were conducted in collaboration with research institutions and specialized clinics, where motion analysis was not only used for research purposes but was actually integrated as part of clinical trials [49,54,61,62].

In conclusion, gait analysis combined with DL techniques can be helpful in understanding and interpreting gait abnormalities, enabling the prediction of PD and disease severity, and discriminating between different neurodegenerative diseases. However, the choice of the best DNN architecture may depend on the size of the dataset and the specific goals of the research or application. In general, larger and more complex datasets may benefit from more sophisticated architectures, such as deeper or more advanced models that can capture intricate patterns in the data. Conversely, simpler architectures may be sufficient for smaller or less complex datasets, which suggests that researchers should consider factors such as the dataset size, data complexity, and the specific goals of their study when selecting an appropriate DNN architecture. Additionally, it could be essential to compare different DL techniques applied to the same dataset to determine the most effective approach for achieving the desired outcomes. Such comparisons can provide valuable insights into the relative performances of various techniques to help identify the best methods for specific tasks. By evaluating and contrasting these techniques on identical datasets, researchers can make more informed decisions and enhance the overall effectiveness of their DL applications. However, it is also important to recognize that not all studies may justify such comparisons. Some studies might have specific objectives or constraints that limit the applicability of direct comparisons, which makes it crucial to consider these factors when interpreting the results.

## Figures and Tables

**Table 1:** Studies employing Wearable Sensors.

Reference	Aim of the Study	Type of Dataset	Number of Subjects (Female\Male)	Mean Age of the Subjects	Acquisition Device	Features	Analytical Methods	Main Results
Abujrida et al., 2023, [46]	Remotely identify the adherence of PD patients to prescribed therapy through DeepaMed, which is a smartphone-based DL approach that distinguishes patients’ gait before and after therapy.	Private	154\302	PD: 63.57 ± 8.09, HC: 40.14 ± 15.45	Smarphone accelerometer and gyroscope sensors	Time, frequency, statistical, and wavelet domain features	DeepaMed CNN	Accuracy: 98.2%; precision: 97.7%; recall: 97.7%; F1: 98.0%
Peraza et al., 2021, [49]	Automatic gait analysis using wearable sensors that leverage triaxial accelerometry and extract gait parameters from four sensors. The detection of gait events is based on DL algorithms (U-Net).	Private	12	64.2	Two AX3 and two GENEActiv sensors	Spatio-temporal gait features	CNN (U-Net), two-layer DNN	Pearson correlations coefficients > 0.75 for the space parameters. The stance time and stride time were significantly different for the normal gait task
Erdaş et al., 2023, [50]	Detect and assess the severity of PD, HD, and ALS using gait data and dynamics through various ML methods, including pure ML and the one-dimensional CNN method, along with ensemble techniques, like voting and stacking to enhance overall performance.	Public (The Gait Dynamics in Neuro-Degenerative Disease Database of PhysioNet)	36\28	HC 47, PD 61.5, HD 54, ALS 53	One ground force sensor on each foot	Kinematic measures	Multi-layer perceptron, random forest, extra trees, and k-nearest neighbor as classification; voting and stacking, and 1-dimensional CNN as regression	Random forest: accuracy 58.61%; precision 58.42%; recall 58.49%; F1 58.45% 1D-CNN: Accuracy 68.11%; Precision 69.05%; Recall 68.16%; F1 67.77%
Lin et al., 2020, [51]	Develop aDL-based algorithm (CNN) for detecting NDD disorders (PD, HD, ALS) using a recurrence plot derived from vertical ground reaction force signals.	Public (The Gait Dynamics in Neuro-Degenerative Disease Database of PhysioNet)	36\28	HC 47, PD 61.5, HD 54, ALS 53	One ground force sensor on each foot	Time–frequency spectrograms	AlexNet CNN	Accuracy > 95%; sensitivity > 90%; specificity > 90%; AUC > 90%
Setiawan et al., 2021, [52]	Develop an artificial intelligence-based algorithm (CNN) for detecting NDD (PD, HD, ALS) using a time–frequency spectrograms derived from vertical ground reaction force signals.	Public (The Gait Dynamics in Neuro-Degenerative Disease Database of PhysioNet)	36\28	HC 47, PD 61.5, HD 54, ALS 53	One ground force sensor on each foot	Time–frequency spectrograms	AlexNet CNN	Accuracy > 90%; Sensitivity > 80%; Specificity > 90%; AUC > 60%
Vásquez-Correa et al., 2019, [53]	Multimodal analysis of motor abilities in patients with PD through the use of DL architectures based on TFR and CNN by integrating information from vocal, writing, and gait signals. The proposed method aimed to model the difficulty patients face in initiating and stopping movements of the upper and lower limbs, as well as in language.	Private	47\36	54.5	eGaIT system	Time–frequency spectrograms and Spatio-temporal features	Individual CNNs are trained for each modality	Accuracy: 97.6%; AUC: 98.8% (with the fusion of the three bio-signals)
Carvajal et al., 2022, [54]	Classify subjects with PD compared with HCs using three different DL architectures: CNN, GRU, and a combination of CNN and GRU, which are considered state-of-the-art in gait analysis. Two subgroups of HC were included: elderly (EHC) and young (YHC).	Private	68\66	52	eGaIT system	Spatio-temporal gait features (segment of 3 s of raw time series)	CNN, GRU, and CNN + GRU	Accuracy: CNN 82.7% (YHC group), 82.4% (EHC group); 82.7% (classification of PD vs. EHC), 92.1% (classification of PD vs. YHC); CNN and GRU 83.7%; 92.7% (classification of PD vs. EHC/YHC) Sensitivity > 70%; specificity > 72%; AUC > 80%
Ma et al., 2023, [55]	Develop of an explainable learning architecture (XGBoost and CNN) that integrates mechanisms of DL and ML, including data selection, feature evaluation, and data balancing, for gait detection in patients with PD.	Public (Ga, Ju, and Si datasets)	68\98	63.3	8 ground force sensor on each foot	The force domain, the peak domain (mean, standard deviation, max and min value of peak data) and the abnormality domain	XGBoost and CNN	XGBoost: 97.32%; CNN: 98.4%
Zhong et al., 2023, [56]	Develop of a robust and innovative graphical adaptive network based on the frequency domain (RFdGAD) to identify PD through gait information, specifically vertical ground reaction force signals recorded by foot sensors.	Public (Ga, Ju, and Si datasets)	68\98	117 < 70 yo, 49 > 70 yo	8 ground force sensor on each foot	Time and frequency domain features	RFdGAD	Accuracy > 75%, F1 > 70%
Aşuroğlu et al., 2022, [57]	Develop a hybrid DL model to predict the severity of PD. In this combined DL approach, the temporal and frequency features of ground reaction force sensors are converted and used as input for the CNN + LWRF architecture.	Public (Physionet Gait in Parkinson’s Disease)	68\98	63.3	8 ground force sensor on each foot	Time and frequency domain features	CNN and LWRF	Accuracy: 99.5%; Sensitivity: 98.7%; Specificity: 99.1%
Setiawan et al., 2021, [58]	Develop an innovative algorithm for detecting and classifying the severity of PD using DL approaches and relying on signals of vertical ground reaction force. Various types of CNNs were employed as classifiers.	Public (Ga, Ju, and Si datasets)	68\98	63.3	8 ground force sensor on each foot	Time–frequency spectrograms	CNN, AlexNet, ResNet-50, ResNet-101, and GoogLeNet	Multi-class classification: accuracy 98.16%, 98.24%, 98.27% (Ga, Ju, Si datasets); sensitivity 98.15%, 98.06%, 97.73% (Ga, Ju, Si datasets), specificity 98.16%, 98.38%, 98.76% (Ga, Ju, Si datasets), AUC 98% (Ga, Ju, Si datasets); Two-class classification: accuracy 99.11%, 99.01%, 98.56% (Ga, Ju, Si datasets); sensitivity 99.77%, 98.94%, 98.85% (Ga, Ju, Si datasets), specificity 98.80%, 99.04%, 98.41% (Ga, Ju, Si datasets), AUC 99% (Ga, Ju, Si datasets)
Xia et al., 2020, [59]	Implement of a gait assessment method to provide a binary classification between PD-associated and normal walks, as well as the severity level of the disease. The proposed system adopts a dual-modal model based on DL, where both left and right walks are separately modeled using a CNN, followed by a LSTM network.	Public (Ga, Ju, and Si datasets)	68\98	63.3	8 ground force sensor on each foot	Force vs. time curve	CNN-LSTM	Predict PD gaits (Ga dataset): accuracy 99.31, sensitivity 99.35%; specificity 99.23%; Classify PD patients with different H&Y scores (Si dataset): accuracy 99.01%
El Maachi et al., 2020, [60]	Develop an advanced PD detection system based on DL techniques to analyze gait information. The approach of 1D-CNN was adopted to build a classifier. The model processes 18 1D vertical ground reaction force signals from foot sensors.	Public (Physionet Gait in Parkinson’s Disease)	68\98	63.3	8 ground force sensor on each foot	Spatio-temporal gait features	1D-ConvNet	Predict PD: accuracy 98.7%; sensitivity 98.1%; specificity 100%; Predict Parkinson’s severity: accuracy 85.3%; precision 87.3%

PD: Parkinson’s Disease; DL: Deep Learning; ML: Machine Learning; CNN: Convolutional Neural Network; HD: Huntington’s Disease; ALS: Amyotrophic Lateral Sclerosis; HC: Healthy Control; LWRF: LocallyWeighted Random Forests; NDD: Neurodegenerative Diseases.

**Table 2:** Studies employing Video Capture.

Reference	Aim of the Study	Type of Dataset	Number of Subjects (Female\Male)	Mean Age of the Subjects	Acquisition Device	Features	Analytical Methods	Main Results
Eguchi et al., 2023, [61]	Propose a CNN to estimate UPDRS severity scores and subscores of axial symptoms, bradykinesia, rigidity, and tremor.	Private	44\30	63.4 ± 8.2	Video camera	Spatio-temporal gait features	ECO-Lite CNN	The goodness of the model the coefficient of determination was evaluated. In particular, axial symptoms, bradykinesia, rigidity, and tremor: 0.59, 0.77, 0.56, and 0.46, respectively
Rupprechter et al., 2021, [62]	Investigate a markerless motion capture system using videos as a component of routine gait assessments to evaluate the motor performances of PD patients.	Private	Not specified	Not specified	Video camera	Spatio-temporal gait features and arm swing	OpenPose	Correlation coefficient 0.80
Zanela et al., 2022, [63]	Evaluate gait impairments and assessing the disease burden by employing human estimation pose system OpenPose and a stereoscopic device.	Private	4\6	62.7 ± 13.2	Video camera	Spatial coordinates	OpenPose	The authors demonstrated good effectiveness of the proposed system in extracting the main features concerning the PD patients’ gaits
Abe et al., 2022, [64]	Investigate the peak-to-peak data regarding the left and right arm swing in PD patients using OpenPose-based gait analysis and video acquired by a smartphone camera.	Private	28 (6\13 PD)	not specified	Smartphone or consumer video camera	P-P (peak-to-peak) Left, P-P right, ASA (arm swing asymmetry)	OpenPose	P-P = 72.7% and ASA = 82.4% of accuracy, respectively
Guayacán et al., 2022, [65]	Proposing a markerless strategy, DensePose CNN, for the analysis of body segment kinematics to obtain PD characterisation during walking, captured in sagittal video sequences using a single camera.	Private	10\12	72.3 ± 7.4	Videocamera	Kinematic measures	DensePose	Accuracy: 99.6% for lower-limbs
Zhang et al., 2023, [66]	Propose a method for recognising the gait of PD patients. First, skeletal features were extracted from the videos using OpenPose. Then, they used a weighted adjacency matrix with virtual connection and multi-scale temporal convolution in a spatiotemporal graph convolution network graph convolutional neural network (WM-STGCN), which provides an efficient mechanism for direct learning of joint trajectories.	Private	50	Not specified	Smartphone accelerometer and gyroscope sensors (Samsung)	Spatio-temporal gait features	OpenPose + WM–STGCN	Accuracy: 87.1%, sensitivity: 86.7%, specificity: 87.5%, precision: 86.7%
Michael H. Li et al., 2018, [67]	Propose a DL-based pose estimation algorithm for a CPM for extracting 15 gait kinematic features in order to train an ML model for detecting and estimating the severity of levodopa-induced dyskinesia and parkinsonisms.	Private	9 (4\5)	64	A single camera	Kinematic measures	CPM and random forest classifier	Unified Dyskinesia Rating Scale (UDysRS) and Unified Parkinson’s Disease Rating Scale (UPDRS) scores were predicted with r = 0.741 and 0.530, respectively
Sato et al., 2019, [68]	Propose a method for quantifying gait features and detecting FOG events by extracting the cadence from normal and parkinsonian gait movies recorded with a home video camera.	Public (CASIA Dataset-B)	119	HC 20, PD 65	Video camera	Spatio-temporal gait features	OpenPose	Comparison between the cadence laterally viewed movie and the frontally viewed movie of the same gait. Good consistency between them: R = 0.754, RMSE = 7.24, and MAE = 6.05
Hu et al., 2019, [69]	Propose a promised novel graph convolutional neural network (GCNN) using the FoG detection method.	Private	45	Not specified	Video camera	Spatial-temporal gait features	GCNN	Accuracy > 87%, sensitivity > 80%, specificity > 79%, AUC > 0.80
Kaur et al., 2022, [70]	Investigate the effectiveness of a vision-based model for classifying gait strides in persons with different neurological disorders. By segmenting the gait steps and identifying heel strikes, several DL algorithms, such as CNN and RNN, were trained.	Private	14\19	66 ± 5 MS, 68 ± 9 PD, 63 ± 9 HC	Video camera	Segmentation of the gait steps and heel strike	4 convolutional architectures (CNN, ResNet, MSResNet, TCN), 3 recurrent architectures (RNN, LSTM, GRU)	In single task, the RNN resulted in the highest accuracy and AUC of 78.1% and 0.87, and the CNN had highest accuracy of 75% in dual trials.
Gül et al., 2023, [71]	Propose a hybrid system based on CNN and use videos of 28 patients taken from front, back, and both sides during walking in order to distinguish different neurological disorders.	Private	28	Not specified	Video camera	Joint coordinates	CNN	Accuracy, sensitivity, specificity > 80%
Iseki et al., 2023, [72]	Propose a markerless motion capture system (Three-Dimensional Pose Tracker for Gait Test (TDPT-GT)) based on ML combined with an iPhone camera to distinguish a pathological gait from a control gait.	Private	131\143	PD: 74.5 ± 7.8, HC: 72.9 ± 11.1	TDPT-GT (iPhone camera)	Spatio-temporal gait features	Light GBM	Accuracy > 70%; sensitvity > 63%, specificity > 72%, AUC > 0.77

PD: Parkinson’s Disease; DL: Deep Learning; ML: Machine Learning; CNN: Convolutional Neural Network; HC: Healthy Control; MS: Multiple Sclerosis; RNN: Recurrent Neural Network; FOG: Freezing Of Gait; CPM: Convolutional Pose Machines.

## Data Availability

No new data were created.

## Supplementary Materials

The following supporting information can be downloaded at: https://www.mdpi.com/article/10.3390/s24185957/s1.

## Abbreviations

The following abbreviations are used in this manuscript:

ALS	amyotrophic lateral sclerosis
ARR	Anova with Recursive Reduction
ASA	arm swing asymmetry
AUC	Area Under the Curve
CNN	Convolutional Neural Network
CPM	Convolutional Pose Machine
DL	Deep Learning
DNN	Deep Neural Network
FoG	Freezing of Gait
FRL	frequency representation learning
GAD	graph adaptive network block
GCNN	graph convolutional neural network
GRU	Gated Recurrent Unit
HC	healthy control
HD	Huntington’s Disease
HPE	Human Pose Estimation
LSTM	long short-term memory
MDS-UPDRS	Movement Disorder Society-Unified Parkinson’s Disease Rating Scale
ML	Machine Learning
MS	Multiple Sclerosis
NDD	neurodegenerative disease
PD	Parkinson’s Disease
P-P	peak-to-peak
PRISMA	Preferred Reporting Items for Systematic Reviews and Meta-Analyses
RFdGAD	robust frequency-domain-based graph adaptive network
RNN	Recurrent Neural Network
TDPT-GT	Three-Dimensional Pose Tracker for Gait Test
vGRF	Vertical Ground Reaction Force
WM-STGCN	Weighted adjacency matrix with virtual connection and Multi-scale temporal convolution in a Spatiotemporal Graph Convolution Networkgraph convolutional neural network

## References

[1] de Lau L.M., Breteler M.M. (2006). Epidemiology of Parkinson’s disease. Lancet Neurol..

[2] Twelves D., Perkins K.S.M., Counsell C. (2002). Systematic review of incidence studies of Parkinson’s disease. Mov. Disord..

[3] Balestrino R., Schapira A. (2020). Parkinson disease. Eur. J. Neurol..

[4] Postuma R.B., Berg D., Stern M.B., Poewe W., Olanow C.W., Oertel W.H., Obeso J.A., Marek K., Litvan I., Lang A.E. (2015). MDS clinical diagnostic criteria for Parkinson’s disease. Mov. Disord..

[5] Mirelman A., Bonato P., Camicioli R., Ellis T.D., Giladi N., Hamilton J.L., Hass C.J., Hausdorff J.M., Pelosin E., Almeida Q.J. (2019). Gait impairments in Parkinson’s disease. Lancet Neurol..

[6] Ba F., Obaid M., Wieler M., Camicioli R., Martin W.W. (2016). Parkinson disease: The relationship between non-motor symptoms and motor phenotype. Can. J. Neurol. Sci..

[7] Dadar M., Gee M., Shuaib A., Duchesne S., Camicioli R. (2020). Cognitive and motor correlates of grey and white matter pathology in Parkinson’s disease. NeuroImage Clin..

[8] Gelb D.J., Oliver E., Gilman S. (1999). Diagnostic criteria for Parkinson disease. Arch. Neurol..

[9] Movement Disorder Society Task Force on Rating Scales for Parkinson’s Disease (2003). The unified Parkinson’s disease rating scale (UPDRS): Status and recommendations. Mov. Disord..

[10] Baker R. (2006). Gait analysis methods in rehabilitation. J. Neuroeng. Rehabil..

[11] Wren T.A., Gorton G.E., Ounpuu S., Tucker C.A. (2011). Efficacy of clinical gait analysis: A systematic review. Gait Posture.

[12] Kulig K., Burnfield J.M. (2008). The role of biomechanics in orthopedic and neurological rehabilitation. Acta Bioeng. Biomech..

[13] Oung Q.W., Muthusamy H., Basah S.N., Lee H., Vijean V. (2017). Empirical Wavelet transform based features for classification of Parkinson’s disease severity. J. Med. Syst..

[14] Sofuwa O., Nieuwboer A., Desloovere K., Willems A.M., Chavret F., Jonkers I. (2005). Quantitative gait analysis in Parkinson’s disease: Comparison with a healthy control group. Arch. Phys. Med. Rehabil..

[15] Pistacchi M., Gioulis M., Sanson F., De Giovannini E., Filippi G., Rossetto F., Marsala S.Z. (2017). Gait analysis and clinical correlations in early Parkinson’s disease. Funct. Neurol..

[16] Amboni M., Ricciardi C., Picillo M., De Santis C., Ricciardelli G., Abate F., Tepedino M.F., D’Addio G., Cesarelli G., Volpe G. (2021). Gait analysis may distinguish progressive supranuclear palsy and Parkinson disease since the earliest stages. Sci. Rep..

[17] Russo M., Amboni M., Barone P., Pellecchia M.T., Romano M., Ricciardi C., Amato F. (2023). Identification of a Gait Pattern for Detecting Mild Cognitive Impairment in Parkinson’s Disease. Sensors.

[18] Russo M., Ricciardi C., Amboni M., Volzone A., Barone P., Romano M., Francesco A. (2023). A Cluster Analysis for Parkinson’s Disease Phenotyping with Gait Parameters. Proceedings of the 2023 IEEE International Conference on Metrology for eXtended Reality, Artificial Intelligence and Neural Engineering (MetroXRAINE).

[19] Morris M., Iansek R., Matyas T., Summers J. (1998). Abnormalities in the stride length-cadence relation in parkinsonian gait. Mov. Disord. Off. J. Mov. Disord. Soc..

[20] Amboni M., Iuppariello L., Iavarone A., Fasano A., Palladino R., Rucco R., Picillo M., Lista I., Varriale P., Vitale C. (2018). Step length predicts executive dysfunction in Parkinson’s disease: A 3-year prospective study. J. Neurol..

[21] Ferreira F., Gago M.F., Bicho E., Carvalho C., Mollaei N., Rodrigues L., Sousa N., Rodrigues P.P., Ferreira C., Gama J. (2019). Gait stride-to-stride variability and foot clearance pattern analysis in Idiopathic Parkinson’s Disease and Vascular Parkinsonism. J. Biomech..

[22] Connie T., Aderinola T.B., Ong T.S., Goh M.K.O., Erfianto B., Purnama B. (2022). Pose-Based Gait Analysis for Diagnosis of Parkinson’s Disease. Algorithms.

[23] Cimolin V., Galli M. (2014). Summary measures for clinical gait analysis: A literature review. Gait Posture.

[24] Dicharry J. (2010). Kinematics and kinetics of gait: From lab to clinic. Clin. Sport. Med..

[25] Abate F., Russo M., Ricciardi C., Tepedino M.F., Romano M., Erro R., Pellecchia M.T., Amboni M., Barone P., Picillo M. (2023). Wearable sensors for assessing disease severity and progression in Progressive Supranuclear Palsy. Park. Relat. Disord..

[26] Pisani N., Ricciardi C., Picillo M., Abate F., Avallone A.R., Amato F., Cesarelli M. Using Wearable Sensors and Motion Parameters for Recognizing Progressive Supranuclear Palsy Phenotypes. Proceedings of the 2023 IEEE International Conference on Metrology for eXtended Reality, Artificial Intelligence and Neural Engineering (MetroXRAINE).

[27] Ricciardi C., Pisani N., Donisi L., Abate F., Amboni M., Barone P., Picillo M., Cesarelli M., Amato F. (2023). Agreement between Optoelectronic System and Wearable Sensors for the Evaluation of Gait Spatiotemporal Parameters in Progressive Supranuclear Palsy. Sensors.

[28] Prisco G., Romano M., Esposito F., Cesarelli M., Santone A., Donisi L., Amato F. (2024). Capability of Machine Learning Algorithms to Classify Safe and Unsafe Postures during Weight Lifting Tasks Using Inertial Sensors. Diagnostics.

[29] Muro-De-La-Herran A., Garcia-Zapirain B., Mendez-Zorrilla A. (2014). Gait analysis methods: An overview of wearable and non-wearable systems, highlighting clinical applications. Sensors.

[30] Gunduz H. (2019). Deep learning-based Parkinson’s disease classification using vocal feature sets. IEEE Access.

[31] Wang W., Lee J., Harrou F., Sun Y. (2020). Early detection of Parkinson’s disease using deep learning and machine learning. IEEE Access.

[32] Caliskan A., Badem H., Basturk A., Yuksel M. (2017). Diagnosis of the parkinson disease by using deep neural network classifier. IU-J. Electr. Electron. Eng..

[33] Landolfi A., Ricciardi C., Donisi L., Cesarelli G., Troisi J., Vitale C., Barone P., Amboni M. (2021). Machine Learning Approaches in Parkinson’s Disease. Curr. Med. Chem..

[34] Ricciardi C., Amboni M., De Santis C., Ricciardelli G., Improta G., Cesarelli G., D’Addio G., Barone P. Classifying patients affected by Parkinson’s disease into freezers or non-freezers through machine learning. Proceedings of the 2020 IEEE International Symposium on Medical Measurements and Applications (MeMeA).

[35] Sivaranjini S., Sujatha C. (2020). Deep learning based diagnosis of Parkinson’s disease using convolutional neural network. Multimed. Tools Appl..

[36] Alharthi A.S., Yunas S.U., Ozanyan K.B. (2019). Deep learning for monitoring of human gait: A review. IEEE Sensors J..

[37] Horst F., Lapuschkin S., Samek W., Müller K.R., Schöllhorn W.I. (2019). Explaining the unique nature of individual gait patterns with deep learning. Sci. Rep..

[38] Albuquerque P., Machado J.P., Verlekar T.T., Correia P.L., Soares L.D. (2021). Remote GAIT type classification system using markerless 2D video. Diagnostics.

[39] Kondragunta J., Wiede C., Hirtz G. (2019). Gait analysis for early Parkinson’s disease detection based on deep learning. Curr. Dir. Biomed. Eng..

[40] Sibley K.G., Girges C., Hoque E., Foltynie T. (2021). Video-based analyses of Parkinson’s disease severity: A brief review. J. Park. Dis..

[41] Park S.H., Seo N.Y., Hwang S.M., Park H.Y., Jung Y.J. (2022). Quantifying Finger-tapping-test Scores using a Three-dimensional Motion Analysis Program: A Preliminary Study. J. Magn..

[42] Shin J.H., Yu R., Ong J.N., Lee C.Y., Jeon S.H., Park H., Kim H.J., Lee J., Jeon B. (2021). Quantitative gait analysis using a pose-estimation algorithm with a single 2D-video of Parkinson’s disease patients. J. Park. Dis..

[43] Stenum J., Rossi C., Roemmich R.T. (2021). Two-dimensional video-based analysis of human gait using pose estimation. PLoS Comput. Biol..

[44] Page M.J., McKenzie J.E., Bossuyt P.M., Boutron I., Hoffmann T., Mulrow C.D., Shamseer L., Tetzlaff J., Akl E.A., Brennan S. (2021). The PRISMA 2020 statement: An updated guideline for reporting systematic reviews. BMJ.

[45] Liberati A., Altman D.G., Tetzlaff J., Mulrow C., Gøtzsche P.C., Ioannidis J.P., Clarke M., Devereaux P.J., Kleijnen J., Moher D. (2009). The PRISMA statement for reporting systematic reviews and meta-analyses of studies that evaluate health care interventions: Explanation and elaboration. BMJ.

[46] Abujrida H., Agu E., Pahlavan K. (2023). DeepaMed: Deep learning-based medication adherence of Parkinson’s disease using smartphone gait analysis. Smart Health.

[47] Chen F., Chen S., Si A., Luo Y., Hu W., Zhang Y., Ma J. (2022). The long-term trend of Parkinson’s disease incidence and mortality in China and a Bayesian projection from 2020 to 2030. Front. Aging Neurosci..

[48] Picillo M., Nicoletti A., Fetoni V., Garavaglia B., Barone P., Pellecchia M.T. (2017). The relevance of gender in Parkinson’s disease: A review. J. Neurol..

[49] Peraza L.R., Kinnunen K.M., McNaney R., Craddock I.J., Whone A., Morgan C., Joules R., Wolz R. (2021). An Automatic Gait Analysis Pipeline for Wearable Sensors: A Pilot Study in Parkinson’s Disease. Sensors.

[50] Erdaş Ç.B., Sümer E., Kibaroğlu S. (2023). Neurodegenerative diseases detection and grading using gait dynamics. Multimed. Tools Appl..

[51] Lin C., Wen T.C., Setiawan F. (2020). Evaluation of vertical ground reaction forces pattern visualization in neurodegenerative diseases identification using deep learning and recurrence plot image feature extraction. Sensors.

[52] Setiawan F., Lin C.W. (2021). Identification of neurodegenerative diseases based on vertical ground reaction force classification using Time–Frequency Spectrogram and deep learning neural network features. Brain Sci..

[53] Vásquez-Correa J.C., Arias-Vergara T., Orozco-Arroyave J.R., Eskofier B., Klucken J., Nöth E. (2019). Multimodal Assessment of Parkinson’s Disease: A Deep Learning Approach. IEEE J. Biomed. Health Inform..

[54] Carvajal-Castaño H.A., Pérez-Toro P.A., Orozco-Arroyave J.R. (2022). Classification of Parkinson&rsquo;s Disease Patients&mdash;A Deep Learning Strategy. Electronics.

[55] Ma Y.W., Chen J.L., Chen Y.J., Lai Y.H. (2021). Explainable deep learning architecture for early diagnosis of Parkinson’s disease. Soft Comput..

[56] Zhong C., Ng W.W.Y. (2023). A Robust Frequency-Domain-Based Graph Adaptive Network for Parkinson’s Disease Detection from Gait Data. IEEE Trans. Multimed..

[57] Aşuroğlu T., Oğul H. (2022). A deep learning approach for parkinson’s disease severity assessment. Health Technol..

[58] Setiawan F., Lin C.W. (2021). Implementation of a deep learning algorithm based on vertical ground reaction Force Time–Frequency features for the detection and severity classification of Parkinson’s disease. Sensors.

[59] Xia Y., Yao Z., Ye Q., Cheng N. (2020). A Dual-Modal Attention-Enhanced Deep Learning Network for Quantification of Parkinson’s Disease Characteristics. IEEE Trans. Neural Syst. Rehabil. Eng..

[60] Maâchi I.E., Bilodeau G.A., Bouachir W. (2020). Deep 1D-Convnet for accurate Parkinson disease detection and severity prediction from gait. Expert Syst. Appl..

[61] Eguchi K., Takigawa I., Shirai S., Takahashi-Iwata I., Matsushima M., Kano T., Yaguchi H., Yabe I. (2023). Gait video-based prediction of unified Parkinson’s disease rating scale score: A retrospective study. BMC Neurol..

[62] Rupprechter S., Morinan G., Peng Y., Foltynie T., Sibley K., Weil R.S., Leyland L.A., Baig F., Morgante F., Gilron R. (2021). A clinically interpretable computer-vision based method for quantifying gait in parkinson’s disease. Sensors.

[63] Zanela A., Schirinzi T., Mercuri N.B., Stefani A., Romagnoli C., Annino G., Bonaiuto V., Cerroni R. (2022). Using a video device and a deep learning-based pose estimator to assess gait impairment in neurodegenerative related disorders: A pilot study. Appl. Sci..

[64] Abe K., Tabei K.I., Matsuura K., Kobayashi K., Ohkubo T. (2022). Relationship Between the Results of Arm Swing Data From the OpenPose-Based Gait Analysis System and MDS-UPDRS Scores. IEEE Access.

[65] Guayacán L.C., Manzanera A., Martínez F. (2022). Quantification of parkinsonian kinematic patterns in body-segment regions during locomotion. J. Med Biol. Eng..

[66] Zhang J., Lim J., Kim M.H., Hur S., Chung T.M. (2023). WM–STGCN: A novel spatiotemporal modeling method for Parkinsonian gait recognition. Sensors.

[67] Li M.H., Mestre T.A., Fox S.H., Taati B. (2018). Vision-based assessment of parkinsonism and levodopa-induced dyskinesia with pose estimation. J. Neuroeng. Rehabil..

[68] Sato K., Nagashima Y., Mano T., Iwata A., Toda T. (2019). Quantifying normal and parkinsonian gait features from home movies: Practical application of a deep learning–based 2D pose estimator. PLoS ONE.

[69] Hu K., Wang Z., Mei S., Martens K.A.E., Yao T., Lewis S.J., Feng D.D. (2019). Vision-based freezing of gait detection with anatomic directed graph representation. IEEE J. Biomed. Health Inform..

[70] Kaur R., Motl R.W., Sowers R., Hernandez M.E. (2022). A Vision-Based Framework for Predicting Multiple Sclerosis and Parkinson’s Disease Gait Dysfunctions—A Deep Learning Approach. IEEE J. Biomed. Health Inform..

[71] Gül S., Soylu E., Terzi M., Türkoğlu M., Koca K.A. (2023). Making the Discrimination in the Walking Parameters of Individuals with Multiple Sclerosis and Parkinson’s Disease with Machine Learning. Turk. J. Neurol. Noroloji Derg..

[72] Iseki C., Hayasaka T., Yanagawa H., Komoriya Y., Kondo T., Hoshi M., Fukami T., Kobayashi Y., Ueda S., Kawamae K. (2023). Artificial Intelligence Distinguishes Pathological Gait: The Analysis of Markerless Motion Capture Gait Data Acquired by an iOS Application (TDPT-GT). Sensors.

[73] Mei J., Desrosiers C., Frasnelli J. (2021). Machine learning for the diagnosis of Parkinson’s disease: A review of literature. Front. Aging Neurosci..

[74] Kubota K.J., Chen J.A., Little M.A. (2016). Machine learning for large-scale wearable sensor data in Parkinson’s disease: Concepts, promises, pitfalls, and futures. Mov. Disord..

[75] Shetty S., Rao Y. (2016). SVM based machine learning approach to identify Parkinson’s disease using gait analysis. Proceedings of the 2016 International conference on inventive computation technologies (ICICT).

[76] Trabassi D., Serrao M., Varrecchia T., Ranavolo A., Coppola G., De Icco R., Tassorelli C., Castiglia S.F. (2022). Machine learning approach to support the detection of Parkinson’s disease in IMU-based gait analysis. Sensors.

[77] Abdulhay E., Arunkumar N., Narasimhan K., Vellaiappan E., Venkatraman V. (2018). Gait and tremor investigation using machine learning techniques for the diagnosis of Parkinson disease. Future Gener. Comput. Syst..

[78] Loh H.W., Hong W., Ooi C.P., Chakraborty S., Barua P.D., Deo R.C., Soar J., Palmer E.E., Acharya U.R. (2021). Application of deep learning models for automated identification of Parkinson’s disease: A review (2011–2021). Sensors.

[79] Ng K.D., Mehdizadeh S., Iaboni A., Mansfield A., Flint A., Taati B. (2020). Measuring GAIT variables using computer vision to assess mobility and fall risk in older adults with dementia. IEEE J. Transl. Eng. Health Med..

[80] Wang F.C., Li Y.C., Kuo T.Y., Chen S.F., Lin C.H. (2021). Real-Time Detection of Gait Events by Recurrent Neural Networks. IEEE Access.

